# GIS Approaches for the Estimation of Residential-Level Ambient PM Concentrations

**DOI:** 10.1289/ehp.9169

**Published:** 2006-06-08

**Authors:** Duanping Liao, Donna J. Peuquet, Yinkang Duan, Eric A. Whitsel, Jianwei Dou, Richard L. Smith, Hung-Mo Lin, Jiu-Chiuan Chen, Gerardo Heiss

**Affiliations:** 1 Department of Health Evaluation Sciences, Pennsylvania State University College of Medicine, Hershey, Pennsylvania; USA; 2 Department of Geography, Pennsylvania State University, College Park, Pennsylvania, USA; 3 Department of Epidemiology; 4 Department of Medicine and; 5 Department of Statistics, University of North Carolina–Chapel Hill, Chapel Hill, North Carolina, USA

**Keywords:** cross-validation, geographic information systems, kriging, particulate air pollution, population-based studies

## Abstract

Spatial estimations are increasingly used to estimate geocoded ambient particulate matter (PM) concentrations in epidemiologic studies because measures of daily PM concentrations are unavailable in most U.S. locations. This study was conducted to *a*) assess the feasibility of large-scale kriging estimations of daily residential-level ambient PM concentrations, *b*) perform and compare cross-validations of different kriging models, *c*) contrast three popular kriging approaches, and *d* ) calculate SE of the kriging estimations. We used PM data for PM with aerodynamic diameter ≤10 μm (PM_10_) and aerodynamic diameter ≤ 2.5 μm (PM2.5) from the U.S. Environmental Protection Agency for the year 2000. Kriging estimations were performed at 94,135 geocoded addresses of Women’s Health Initiative study participants using the ArcView geographic information system. We developed a semiautomated program to enable large-scale daily kriging estimation and assessed validity of semivariogram models using prediction error (PE), standardized prediction error (SPE), root mean square standardized (RMSS), and SE of the estimated PM. National- and regional-scale kriging performed satisfactorily, with the former slightly better. The average PE, SPE, and RMSS of daily PM_10_ semivariograms using regular ordinary kriging with a spherical model were 0.0629, −0.0011, and 1.255 μg/m^3^, respectively; the average SE of the estimated residential-level PM_10_ was 27.36 μg/m^3^. The values for PM2.5 were 0.049, 0.0085, 1.389, and 4.13 μg/m^3^, respectively. Lognormal ordinary kriging yielded a smaller average SE and effectively eliminated out-of-range predicted values compared to regular ordinary kriging. Semiautomated daily kriging estimations and semivariogram cross-validations are feasible on a national scale. Lognormal ordinary kriging with a spherical model is valid for estimating daily ambient PM at geocoded residential addresses.

Large-scale, population-based epidemiologic investigations of the health effects of ambient air pollution often rely on measurements from a network of air quality monitors maintained by the U.S. Environmental Protection Agency (U.S. EPA 1995a, 1995b, 2005). The Air Quality System (AQS) is the only national ambient air pollution database currently available for public use in the United States. The availability of individual-level health outcome and covariable data from national-scale studies that often characterize participants over the course of several years enables researchers to study the acute effects of ambient air pollution using individual-level data (Liao et al. 2004, 2005a; Sullivan et al. 2005; Wellenius et al. 2005; Whitsel et al. 2004). This approach requires measures of daily particulate matter (PM) exposures, ideally assessed as close to the individual level as possible, such as at participant residences or in immediate proximity to participants themselves. Because daily measures of ambient PM concentrations from the AQS are unavailable in the large majority of locations, spatial estimation methods using geographic information systems (GIS) are increasingly being considered to estimate geocoded location-specific ambient PM concentrations, such as kriging methods. Important methodologic and practical issues still need to be resolved, however. This study was designed to *a*) assess the feasibility of large-scale kriging estimation of daily residential-level ambient PM concentrations, *b*) perform and compare cross-validations of different kriging models, *c*) determine and contrast the most appropriate kriging approaches, and *d*) calculate the SEs of the kriging estimations.

## Materials and Methods

We obtained from AQS the PM_10_ and PM_2.5_ (PM with aerodynamic diameter ≤ 10 and 2.5 μm, respectively) data from 1993–2004 (U.S. EPA 2005). The data from 2000 were used for this study after eliminating duplicate records and converting all measures to the same units and denominator. We calculated “monitor-specific” daily averages based on ≥ 18 hourly measures. Monitor-specific daily averages were set to missing for monitors reporting < 18 hourly measures on any given day. If more than one monitor was operating at the same location on a given day, we then computed “site-specific” daily PM_10_ and PM_2.5_ averages by taking the mean of the monitors’ measures. We also obtained the longitude and latitude for each site from the AQS database. These data served as pollutant- and site-specific daily source data for our study (Liao et al. 2005b).

We geocoded 94,135 addresses of Women’s Health Initiative (WHI) Clinical Trial (CT) participant residences and examination sites in the contiguous 48 United States and District of Columbia, after assessing geocoding vendor error (Whitsel et al. 2004, 2005). Daily PM_10_ and PM_2.5_ concentrations and the associated estimation errors (SEs) are estimated at these geographic locations by the Environmental Epidemiology of Arrhythmogenesis in WHI study (Whitsel 2006).

We used ArcView GIS (version 8.3) and its Geostatistical Analyst Extension (ESRI Inc., Redlands, CA) for semivariogram determination and cross-validation and for subsequent spatial estimation of daily location-specific PM concentrations. Three frequently referenced spatial models (spherical, exponential, Gaussian) (Cressie 1993a; Davis 2002) were considered using the weighted least-squares method (Gribov et al. 2004; Jian et al. 1996) to obtain the “optimal” daily semivariogram parameters (range, partial sill, and nugget). Based on the daily semivariograms, we performed ordinary kriging to estimate the daily mean PM concentration and its SE at each of the 94,135 geocoded addresses. Next, we performed the standard cross-validation—an iterative procedure that omits site-specific PM data points one at a time and refits the model using the remaining data to estimate the PM concentration at the site of the omitted observation. We assessed the validity (also termed “goodness of fit”) of each semivariogram using three cross-validation parameters readily available from the ArcView software package: *a*) the average of prediction error (PE), where PE is the average of the difference between the predicted and measured daily PM values at each monitoring site; *b*) the average of standardized prediction error (SPE), where SPE is the PE divided by the SE of estimation across all sites; and *c*) root mean square standardized (RMSS), the standard deviation (SD) of all SPEs across all sites. Additionally, we assessed the goodness of fit of each semivariogram by the average of the SEs of the estimations, generated by the kriging procedure, across all 94,135 geocoded addresses. The expectations for a good-fitting semivariogram and kriging model are an average PE and SPE near 0, an RMSS near 1, and a small SE. If RMSS < 1, there is a tendency toward overestimation of the variance; if > 1, there is a tendency toward underestimation (ESRI Inc. 2001). These criteria were consistently used to guide our model selection processes throughout this study (Liao et al. 2005c).

As an alternative to using the automatically calculated semivariogram (calculated using the weighted least-squares method (Gribov et al. 2004; Jian et al. 1996), one can also manually specify the semivariogram parameters to improve the cross-validation parameters in ArcView. We selected six least satisfactory daily semivariograms throughout year 2000 and manually adjusted the semivariogram parameters to obtain the best achievable average RMSS and SPE (RMSS as close to 1 and average SPE as close to 0 as possible). The cross-validation parameters from the weighted least-squares method–calculated semivariograms were then compared to those of the manually adjusted semivariograms.

We performed daily ordinary krigings on both the original scale (regular ordinary krig-ing) and the lognormal scale (lognormal ordinary kriging) (Cressie 1993b; Johnston 2001) for all WHI CT addresses for the year 2000 and compared the cross-validation parameters between the two kriging procedures. Log-normal ordinary kriging was used because it has the ability to eliminate the negative predicted values, which is a problem in ordinary kriging, especially when the source data contain extreme values.

## Results

### Characteristics of the site-specific daily average PM_10_ and PM_2.5_ concentrations

**Figure 1:**
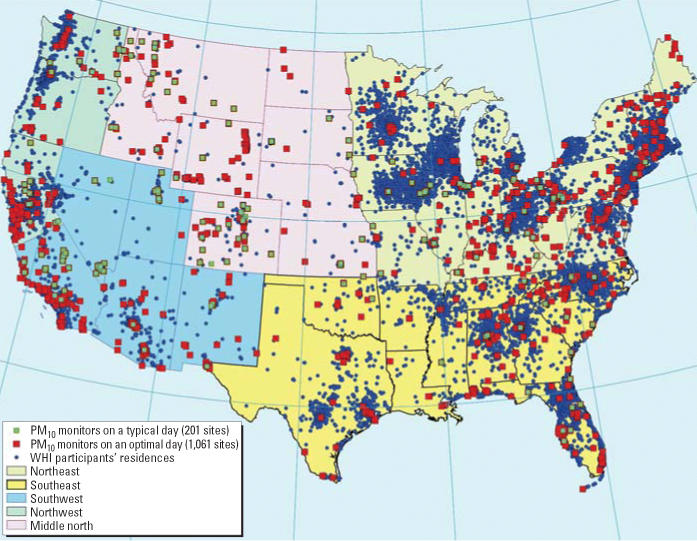
Spatial relationships between the residential locations, PM monitoring sites, and the geographic regions of the study.

During 1994–2003, the number of monitoring sites that provided GIS-usable daily PM_10_ data varied widely (range, 120–1,340). On 17% of days, GIS-usable data were provided by ≥ 400 monitoring sites; on 39% of days, by 200–400 sites; and on 44% of days, by 120–200 sites. The corresponding values for PM_2.5_ during 1999–2003 were 33% of days by ≥ 400 sites and 67% of days by 148–400 sites. Specific to the year 2000, there were averages of 325 PM_10_ and 456 PM_2.5_ monitoring sites operating per day across the contiguous United States, with minima and maxima of 148 and 1,061 sites for PM_10_ and 178 and 1,019 sites for PM_2.5_. As a result, there were 118,791 site-days during 2000 for which we can retrieve measured PM_10_ data and 166,796 site-days for PM_2.5_ data. The mean (± SD) of PM_10_ and PM_2.5_ from these retrievable site-days were 26.29 ± 58.13 and 13.14 ± 8.59 μg/m^3^, respectively, with medians of 21.33 and 11.20 μg/m^3^, respectively. A right-skewed distribution of both PM_10_ and PM_2.5_ are evident, especially for PM_10_. Figure 1 illustrates the spatial relationships between the geocoded addresses and the PM monitoring sites on an optimal day and a typical day. The mean distance between each address and its nearest PM monitor was 12.35 km, with an SD of 13.98 km, a median of 7.81 km, an interquartile range of 10.53 km, and 99th percentile of 68.36 km.

### Comparisons of three widely used spatial models

Tables 1 and 2 present summary statistics of the cross-validation parameters (PE, SPE, and RMSS) comparing three widely used spatial models (spherical, exponential, Gaussian) for PM_10_ and PM_2.5_, respectively. In general, both average PE and average SPE are very close to 0, with a very narrow range of variation from the 366 daily cross-validations. More specifically, > 95% of average PEs were within ± 2 μg/m^3^ of measured PM_10_, and ± 0.5 μg/m^3^ of measured PM_2.5_, an average measurement error that we considered acceptable. In terms of RMSS, we considered > 95% of cross-validations as acceptable, but there were days when RMSS indicated a slight over-or underestimation of the prediction variability. These data support the overall validity of using kriging-based estimation approaches to estimate location-specific PM concentrations across the contiguous United States.

### Comparisons of default and manually adjusted semivariograms

Table 3 presents the cross-validations and actual kriging estimations from the weighted least-squares mean method calculated semivariogram and manually adjusted semivariogram. For the 6 days when the PE, SPE, or RMSS indicated a less satisfactory default-calculated semivariogram, these three cross-validation parameters could be improved satisfactorily through adjustment of the semivariogram parameters by an operator. However, the application of such “improved” semivariograms to the estimation of PM_10_ concentrations at geocoded locations across the United States did not necessarily provide better estimation of location-specific PM (i.e., smaller SEs). To the contrary, the average SEs from the default semivariograms were smaller than those from manually adjusted semivariograms. Because each average SPE of the default-calculated daily semivariograms was close to 0, and each default-calculated daily semivariogram produced a smaller estimation error, we recommend using the default-calculated semivariogram, even though the RMSS from the default-calculated semivariogram was not fully satisfactory.

### Comparisons of regular versus lognormal ordinary krigings

We applied regular ordinary kriging (spherical model, default-calculated daily semivariograms) to estimate daily PM_10_ concentrations at geocoded addresses (*n* = 94,135) of WHI CT participants and examination sites in the contiguous United States. We examined the estimated PM_10_ concentrations and identified 22 days during 2000 when estimated values exceeded the range of observed values. In some cases, the estimated values were negative. The number of addresses affected by this problem ranged from a few on most days to 3.5% of all addresses. This problem was related to skewed PM_10_ distributions and to small numbers of extreme outlying values or operating sites on some days. We therefore compared regular ordinary kriging and log-normal ordinary kriging anticipating that log-normal kriging would attenuate this problem.

Table 4 lists the 22 days on which regular ordinary kriging yielded estimated PM_10_ values that were outside the range of measured values. For comparison, the minima and maxima of the measured and estimated PM_10_ concentrations from both regular and log-normal ordinary krigings are also listed in Table 4. In summary, during 2000, lognor-mal ordinary kriging effectively reduced the number of problematic days from 22 to 1. Even on this one day, lognormal ordinary kriging yielded a minimum value that was closer to the range of measured data than that from regular ordinary kriging.

Table 5 shows the mean values of cross-validation parameters of daily PM_10_ semivariograms for both regular ordinary kriging and lognormal ordinary kriging. Cross-validation parameters were within the acceptable range from both regular and lognormal ordinary krigings, except for the 22 “out-of-range” days as defined above. On these out-of-range days, the SPE was well within the acceptable range for both regular and lognormal krigings, but the RMSS was > 1 from both approaches. Even so, for these out-of-range days RMSS from lognormal ordinary kriging was closer to 1 than that from regular ordinary kriging.

We then performed regular and lognormal ordinary kriging to estimate PM_10_ concentrations at geocoded addresses of WHI CT participants and examination sites, based on year 2000 PM_10_ data (94,135 locations and 366 days). The mean, SD, median, and maximum of the daily mean SE of the estimated PM_10_ from the regular ordinary kriging were 27.36, 83.35, 13.93, and 1160.20 μg/m^3^, respectively. In contrast, those from the lognormal ordinary kriging were 16.29, 6.65, 15.05, and 67.46 μg/m^3^. Clearly, the distribution of the estimation errors from lognormal ordinary kriging was considerably less skewed and had fewer outlying values than that from regular ordinary kriging. Alternative methods (winsorizing extreme PM_10_ values; using ArcView’s “no-sector” option to search for measured data points from a circle centered around a location that needs of an estimation—i.e., disabling the default “sector” search for measured data points in the four sectors of a circle, reducing the range or nugget) were less effective in estimating predicted values within the range of measured values (data not shown).

Similar to the situation observed in PM_10_ estimations, lognormal ordinary kriging also effectively eliminated the negative or out-of-range problem that occurred in about 5% of PM_2.5_ data when using regular ordinary kriging. Other cross-validation parameters were comparable between the lognormal and regular ordinary krigings (data not shown).

### Comparisons between national and regional krigings

From the 61 days when 900 or more monitoring sites were operating in the year 2000 in the 48 contiguous states, the first of such days from each month was selected for comparisons between ordinary kriging models on a national versus regional scale. National krigings and cross-validations were performed on these 12 selected days using daily site-specific PM_10_ data. Regional krigings and cross-validations were performed on the same data using the regional map (Figure 1) that divides the U.S. continent into five regions (northwest, southwest, middle north, southeast, and northeast). These five regions were created based on the assumption that different semivariogram parameters would be needed for different geographic areas. In general, for both regional and national krigings, the average SPE and RMSS from cross-validations of semivariograms calculated for the 12 selected days were very close to 0 and 1, respectively (Table 6)

## Discussion

Classical methods often assume that measures are uniformly or randomly distributed. The assumptions are often inappropriate for analysis of environmental measures because values at neighboring locations are rarely independent, particularly over short distances. This form of dependence (spatial autocorrelation) nonetheless makes it possible to interpolate values at unmonitored locations from known values at monitored locations. Kriging is one such interpolation method originally developed by mining engineers (Krige 1966). It is especially attractive in this setting because it takes the spatial autocorrelation structure function (variogram) into account by considering known values from monitored locations, weighting them with values read from the variogram at corresponding distances, and splitting weights among adjacent locations. The method thereby ensures that interpolations do not depend on monitor density (Legendre and Fortin 1989). By doing so, kriging yields best linear unbiased estimates, in this setting, of location-specific daily mean ambient PM concentrations and their SEs.

Large-scale population-based epidemiologic investigations of the health effects of ambient air pollution often rely on data collected from a network of air quality monitors maintained by the U.S. EPA—the AQS data (U.S. EPA 1995a, 1995b, 2005). It is revealing to compare kriging with interpolation methods used in the well-known time-series and cohort studies of PM effects on mortality and cardiovascular disease (Abbey et al. 1991, 1999; Dockery et al. 1993; Katsouyanni et al. 1996, 2001; Miller et al. 2004, 2005; Pope et al. 2004; Samet et al. 2000a, 2000b). These studies uniformly estimated PM exposures using area-based arithmetic averaging or nearest-neighbor imputation—alternative methods that have important limitations (Moore and Carpenter 1999). Such limitations include the assumption of homogeneous exposures within study areas and the inability (or failure) to estimate exposures or associated PEs. For example, when daily exposure was of interest and there were no operating PM monitors with a study area, data pairs (daily PM concentrations, death counts) were unavailable in these studies. In addition, when longer-term (monthly to yearly) exposure was of interest, area aggregated exposures were based on available measurements within a given time frame. If there were five 24-hr measures in a month, for example, the monthly average exposure was calculated as the mean of the five readings. In contrast, our kriging-based approach estimated daily mean exposures and SEs at geocoded addresses of participants and their examination sites across the contiguous United States that can be readily integrated over time with little influence of missing data. Studies in the geosciences have also found that kriging provides consistently improved interpolation accuracy over traditional inverse-distance weighting and other, simpler spatial interpolation methods (Zimmerman 1999). Another important advantage of GIS-based estimation over the traditional area-average approach is the availability of both the location-specific estimated pollutant concentrations and their SEs.

Our goal in this study was to contribute methodologic and practical insights toward standardized, semiautomated GIS approaches to estimation of daily air pollution concentrations and their associated estimation errors. The air pollution data estimated using these approaches will support the Environmental Epidemiology of Arrhythmogenesis in WHI study (Whitsel 2006) examining the cardiac effects of air pollution in 68,133 post-menopausal women 50–79 years of age at baseline in the WHI CT (WHI Study Group 1998). Here we describe our experience resolving several important methodologic and practical issues in adopting a systematic, standardized, and semiautomated kriging approach to estimate daily air pollution concentrations and the associated estimation errors at geocoded addresses across the contiguous United States over 10 years.

We successfully downloaded from AQS the PM_10_ and PM_2.5_ raw data from 1993–2004. We then cleaned, calculated, and reconstructed site-specific daily PM concentration data ready for GIS applications. It is well known that the monitoring sites in AQS are not randomly distributed, which is one of the assumptions in kriging estimation, and the density of the monitoring sites is relatively low given the size of the contiguous United States. However, the AQS is the only currently available nationwide database. Our cross-validation studies suggest that the AQS data can be used as source data for kriging estimation of ambient pollution concentrations at various locations across the 48 contiguous states.

In this study, we performed cross-validation to assess the goodness of fit of various semivariogram and spatial models using four major parameters: the average PE, SPE, RMSS, and SE of estimation. Details can be found elsewhere (Webster and Oliver 2001), but it is worth noting that in addition to using the SE as a measure of the goodness of fit of a kriging model, one could improve the health effects models by incorporating SE in the models to account for the error in the estimation of location-specific PM concentrations. We consider this an important advantage of GIS-based estimation over the traditional area-average approach and are performing studies of using SE in health effects models.

We compared the performance of three widely spatial models (spherical, exponential, Gaussian) for PM_10_ and PM_2.5_ estimations using regular ordinary kriging on a national scale (Tables 1 and 2). In general, the cross-validation parameters suggest that all three models performed fairly well. Overall, the spherical model seemed to perform slightly better, consistent with the observation that the spatial distribution pattern of ambient air pollutants is closest to the assumption of the spherical model. The spherical model has been used most often in modeling spatially distributed data, providing a further rationale for its use in our large-scale population-based study of the health effects of PM. Furthermore, from the perspective of the cross-validation results, both average PE and average SPE are very close to 0, with a very narrow range of variation from the 366 daily cross-validations. These data support the overall validity of using kriging-based estimation approaches to estimate location-specific PM concentrations across the contiguous United States.

We completed an empirical analysis to investigate whether manually adjusting semivariogram parameters improves *a*) cross-validation parameters and *b*) estimated PM_10_ concentrations and their SEs (Table 3). From these data, we conclude that manually adjusting semivariogram parameters improves cross-validation parameters. However, the application of such “improved” semivariograms to the estimation of PM_10_ concentrations at geocoded locations across the United States did not necessarily provide better estimation of location-specific PM. Therefore, we recommend using the default-calculated semivariogram.

Semivariograms are sensitive to strong positive skewness. As a result, regular ordinary kriging can yield negative predicted values or values exceeding the range of the source data. Kriging works best if the input data have a normal distribution. One solution is to log-transform the input data—using “lognormal kriging.” In the ArcView software package, performing lognormal kriging is a standard option. This option log-transforms the input data to normalize its distribution and attenuate the impact of very large values. It also back-transforms the estimated values and the “unbiased” SE of the estimation to the original scale (Cressie 1993b; Johnston 2001). Our results comparing lognormal ordinary kriging versus regular-scale ordinary kriging suggest that lognormal ordinary kriging not only effectively estimated location-specific PM concentrations within the range of the measured data for the days regular ordinary kriging yielded negative or “out of range” PM estimations, but also yielded a smaller average SE than did regular ordinary kriging and estimations. Therefore, our results support the use of lognormal ordinary kriging as an acceptable solution to the problem commonly posed by positively skewed distributions of environmental data.

Our comparisons of national- versus regional-scale kriging indicate that, in terms of cross-validation results, both performed similarly. However, such comparisons are based on krigings using the source data from optimal days (when > 900 sites across the country were reporting data), which account for only 17% of all days in a year. Therefore, there is additional justification for using national-scale kriging: Usually, there were very few operating sites within a region. On typical days—when only about 200 monitoring sites were operating—ability to derive stable and meaningful semivariograms was greatly impaired. Regional kriging also poses problems for estimation at locations near regional borders. For example, at locations within Washington State but near the Washington–Idaho border, regional kriging is based solely on PM_10_ concentrations in the “Washington/Oregon, Northern California” region. It is not based on PM_10_ concentrations measured immediately across the border in Idaho, despite the real possibility that they would have the largest weights in national-scale kriging estimation. For all these reasons, we recommend national-scale kriging.

Considering the number of study participants and the length of study period (1994–2003) for the Environmental Epidemiology of Arrhythmogenesis in WHI study, development of an automated procedure enabling large-scale daily krigings and semivariogram cross-validations was critical. In this study, we decided to use ArcView for predicting individuals’ PM exposure concentrations because of the flexibility it offers for automation. Because ArcView GIS relies on either the weighted least-squares method or visual adjustment to create semivariograms, we did not compare the relative performance of semivariograms generated using alternative methods such as maximum likelihood and restricted maximum likelihood. For generating semivariograms, we compared only three popular spatial models (spherical, exponential, and Gaussian). Our results, however, do not invalidate alternative spatial models (e.g., power). In the end, we selected the spherical model for our study because it is the most studied model, and its assumption pertaining to the spatial correlation of data is probably closest to our pollutant data. Furthermore, the spherical model seemed to perform as well as or slightly better than the remaining models in terms of cross-validation parameters.

We chose ordinary kriging instead of universal or simple kriging for several reasons. First, the assumption for simple kriging of a known mean concentration on any given day across space is not practical for our data. Although it may seem more appropriate because of the “varying mean” concentration across the contiguous U.S. assumption, universal kriging requires a predetermined set of “exploratory variables” to explain the varying means. The candidates, many of which are spatial variables, include emissions, land use, population, road network distribution, altitude, rainfall, latitude, climatology, and other quality data. Denby et al. (2005) recently recommended a method that uses measured concentration data in combination with some “exploratory variables” as suggested above. However, their approach may not be feasible for a national-scale study such as ours, because little guiding information is available as to how to identify a set of widely acceptable variables that can be applied to the entire nation. Moreover, even if we could identify a set of exploratory variables, we do not know the forms or shapes of their independent and joint relations to the air pollution measures. Further studies that involve large-scale national data using universal kriging are still needed. In this study, we empirically tested whether the non-constant mean assumption for universal kriging was needed; we performed five regional ordinary krigings so that different parts of the country would assume a different mean PM concentration. Our data suggested that regional and national ordinary kriging performed similarly. Therefore, our data indirectly validated and supported the use of national ordinary kriging.

Although the primary objective of our study is to assess the short-term relationship between PM and cardiac responses, the proposed kriging method also enables us to calculate the long-term cumulative exposure of an individual by taking into account the change of his or her residences over time, because the WHI study recorded the residential location history over 10 years. Nevertheless, from the environmental perspective, an inherited limitation of the kriging-based approach is that the estimations of the PM concentrations will provide only surrogates, or the best guesses, of the true exposure levels at the locations of interest. Thus, the accuracy of the estimations depends highly on the quality of the measured data and their spatial correlation. Even if the estimations were made with a high level of confidence, they cannot be directly interpreted as the true individual-level exposures. However, to correlate individual level cardiac responses with a surrogate of location-specific exposure, our approach represents one of the best available methods for a large-scale population-based study.

In summary, our investigation of GIS approaches for estimating daily mean geocoded location-specific air pollutant concentrations and their SEs supports the use of a spherical model to perform lognormal ordinary kriging on a national scale. Our findings also support the use of default-generated semivariograms (estimated using the weighted least-squares method) without visual adjustment. We developed a semiautomated program to access and execute ArcView to implement these approaches for large-scale daily kriging estimations and semivariogram cross-validations. Detailed information about this program can be obtained on request.

## Figures and Tables

**Table 1:** Cross-validation summary statistics and semivariogram parameter estimates for PM_10_ from three different spatial models, year 2000.

Model	Days^a^	Mean	SD	Median	2.5th percentile	97.5th percentile
PE (μg/m^3^)						
Exponential	366	0.2347	1.3212	0.0294	−0.6437	1.6690
Gaussian	366	−0.1097	1.0509	−0.1216	−1.1230	1.0020
Spherical	366	0.0629	1.1999	−0.0705	−0.7914	1.4810
RMSS						
Exponential	366	1.8374	1.5431	1.1410	0.8638	6.0240
Gaussian	366	1.1709	0.9891	1.0070	0.8140	2.2660
Spherical	366	1.2549	0.7988	1.0270	0.8094	4.1550
SPE						
Exponential	366	0.0118	0.0330	0.0036	−0.0274	0.1058
Gaussian	366	−0.0094	0.0333	−0.0071	−0.0418	0.0274
Spherical	366	−0.0011	0.0212	−0.0034	−0.0318	0.0470
Nugget (μg/m^3^)						
Exponential	366	2,837.28	27,839.3	93.5230	0.0000	5,332.40
Gaussian	366	4,096.10	38,738.9	181.975	26.6230	7,466.20
Spherical	366	3,515.02	33,349.0	142.955	0.0000	7,143.10
Partial sill (μg/m^3^)						
Exponential	366	7,957.38	91,589.2	258.515	49.1340	23,007.0
Gaussian	366	6,483.31	73,915.9	176.240	39.4570	23,716.0
Spherical	366	6,374.25	71,024.0	201.215	36.6550	22,736.0
Range (m)						
Exponential	366	2,696,226	2,832,621	1,392,250	282,500	9,064,200
Gaussian	366	2,163,126	2,277,023	1,207,050	262,460	8,958,300
Spherical	366	2,447,936	2,375,933	1,424,050	280,820	8,958,300

a Daily operating monitoring sites range from 148 to 1,061 sites.

**Table 2:** Cross-validation summary statistics and semivariogram parameter estimates for PM_2.5_ from three different spatial models, year 2000.

Model	Days^a^	Mean	SD	Median	2.5th percentile	97.5th percentile
PE (μg/m^3^)						
Exponential	366	0.1067	0.1162	0.0857	−0.0756	0.3835
Gaussian	366	−0.0323	0.0846	−0.0349	−0.2084	0.1187
Spherical	366	0.0491	0.0883	0.0413	−0.1033	0.2571
RMSS						
Exponential	366	2.0953	1.6086	1.4365	0.5974	6.1640
Gaussian	366	0.9562	0.4500	0.9114	0.5517	1.5960
Spherical	366	1.3887	1.3037	1.0014	0.5532	4.5810
SPE						
Exponential	366	0.0253	0.0356	0.0127	−0.0178	0.1097
Gaussian	366	−0.0102	0.0155	−0.0096	−0.0379	0.0178
Spherical	366	0.0085	0.0242	0.0038	−0.0219	0.0749
Nugget (μg/m^3^)						
Exponential	366	9.4120	14.0622	4.2819	0.0000	46.2270
Gaussian	366	26.8536	19.8300	22.2560	3.3694	76.4140
Spherical	366	16.4381	16.5187	12.0995	0.0000	64.1640
Partial sill (μg/m^3^)						
Exponential	366	94.0859	81.4191	70.0215	13.0410	304.610
Gaussian	366	80.2910	102.183	49.9360	8.8309	326.550
Spherical	366	84.3554	82.4740	56.7625	10.1850	299.980
Range (m)						
Exponential	366	4,944,054	3,364,623	4,047,800	758,590	9,064,200
Gaussian	366	3,137,407	2,199,286	2,683,950	564,450	8,904,000
Spherical	366	3,840,664	2,669,710	3,370,250	667,310	8,944,000

a Daily operating monitoring sites range from 178 to 1,019 sites.

**Table 3:** Comparison of estimated PM_10_ (μg/m^3^) at 94,135 geocoded addresses of WHI CT participant residences and examination sites using default and manually modified semivariograms.

	Summary statistics of cross-validations						Summary statistics of estimation					
	PE		RMSS		SPE		Mean PM_10_		Mean SE		PM_10_ difference (default – modified)	
Date	Default	Modified	Default	Modified	Default	Modified	Default	Modified	Default	Modified	Mean	SD
02/16/2000	0.0122	−0.0099	5.034	1.037	0.0470	0.0021	31.19	28.76	9.73	14.02	2.43	3.61
03/05/2000	0.1660	0.0474	5.134	1.360	0.0469	0.0058	20.85	20.10	10.99	13.80	0.75	4.24
07/15/2000	0.5278	0.0193	5.564	1.180	0.0674	−0.0024	24.01	23.83	7.57	10.13	0.18	3.11
08/07/2000	0.5524	−0.1056	6.183	1.134	0.1417	−0.0053	34.84	33.79	14.09	17.27	1.06	2.70
08/19/2000	0.7609	0.3651	4.744	1.146	0.0963	0.0142	25.07	24.76	13.59	13.54	0.30	3.64
10/28/2000	0.4590	0.0363	4.243	1.276	0.0780	0.0018	25.17	24.23	5.57	7.40	0.93	2.16

**Table 4:** Minima and maxima of measured and estimated PM_10_ (μg/m^3^) on the 22 days in 2000 when estimated values exceeded the range of measured values.

		Estimated from ordinary krigings			Estimated from ordinary krigings	
Date	Minimum measured	Regular	Lognormal	Maximum measured	Regular	Lognormal
01/11	3.80	−3.135	5.535	712.00	534.814	261.951
01/15	3.00	2.106	11.756	194.88	162.905	88.078
01/16	3.00	2.102	9.150	167.60	107.460	70.224
02/13	1.00	−4.006	5.938	196.13	100.739	33.147
02/28	3.00	−0.005	7.196	138.50	135.518	77.630
03/05	3.68	−5.278	7.281	186.48	103.308	36.171
03/11	4.00	2.945	9.064	109.15	106.438	42.912
03/18	5.29	3.179	8.841	117.35	108.649	43.124
04/08	4.00	−43.540	9.097	690.00	534.630	78.059
04/16	0.14	−3.759	0.901	171.13	164.973	69.290
05/04	5.65	−5.768	14.550	1063.00	808.397	61.646
05/09	2.00	−15.362	10.889	3059.00	895.213	66.493
05/10	3.00	−18.598	13.805	1513.00	1023.12	252.891
05/14	6.00	5.472	6.175	82.00	79.383	79.051
06/07	9.13	−49.164	18.164	1642.00	1234.99	64.426
06/10	8.00	7.456	8.224	111.79	69.293	74.018
06/15	7.22	5.282	12.582	242.42	235.167	83.429
07/04	7.00	6.946	9.128	90.00	80.347	74.346
08/02	3.00	−1.224	16.587	441.00	356.964	76.597
08/17	8.22	5.296	7.132	200.00	194.675	198.473
08/20	5.00	4.244	5.899	135.00	134.182	83.798
08/30	7.00	6.074	11.696	140.00	112.957	83.781

**Table 5:** Means ± SDs of the cross-validation summary statistics from both ordinary and lognormal krigings, year 2000.

	All days (*n*= 366)		Out-of-range days (*n* = 22)		Within-range days (*n* = 344)	
	SPE	RMSS	SPE	RMSS	SPE	RMSS
Ordinary	−0.0011 ± 0.0212	1.2549 ± 0.7988	0.018489 ± 0.04202	3.329227 ± 1.93762	−0.00147 ± 0.02018	1.18206 ± 0.67478
Lognormal	−0.05012 ± 0.10191	1.390834 ± 1.56927	−0.10918 ± 0.12434	2.374532 ± 2.18070	−0.04635 ± 0.09933	1.327924 ± 1.50445

**Table 6:** Comparisons of goodness of fit between national and regional scale krigings of the 12 days studied in 2000.

	SPE						RMSS					
Date	Natl	SW	NW	MN	SE	NE	Natl	SW	NW	MN	SE	NE
01/01	0.0106	0.0193	−0.0238	0.0008	−0.0168	−0.0125	0.9843	0.9976	1.0042	1.0064	0.9642	0.8617
02/06	0.0034	0.0320	−0.0013	0.0087	0.0241	−0.0126	0.9996	1.0034	1.0203	0.9335	1.0370	0.9816
03/01	0.0159	0.0089	0.0456	0.0062	−0.0079	−0.0215	1.0237	1.0701	1.0505	1.0021	1.0067	1.0397
04/06	−0.0015	0.0038	0.0286	−0.0032	0.0014	0.0140	0.9992	0.9693	1.0927	0.8644	1.0000	0.9995
05/06	−0.0052	0.0284	−0.0420	−0.0075	−0.0095	−0.0178	1.0732	1.0027	1.0162	0.9997	0.9938	0.9361
06/05	−0.0079	0.0150	0.0105	−0.0228	−0.0086	−0.0058	0.9966	0.9694	1.1046	0.9131	1.1005	1.0638
07/05	0.0031	0.0010	0.0083	−0.0571	−0.0233	0.0054	0.9938	0.9052	1.1020	0.9489	1.0043	1.0048
08/04	0.0108	0.0220	−0.0025	0.0069	−0.0208	0.0165	0.9922	0.9990	1.2180	1.0243	0.9932	1.0014
09/03	0.0053	0.0086	−0.0013	−0.0022	0.0054	0.0130	0.9731	1.0328	1.0393	1.0030	0.8441	1.0008
10/03	0.0055	0.0245	0.0164	−0.0314	0.0287	0.0137	0.9692	1.0014	1.0052	0.9925	0.9948	0.9619
11/02	0.0190	0.0565	0.0364	−0.0155	0.0432	0.0080	0.9956	0.9984	0.9210	0.9964	0.9933	1.0103
12/02	0.0130	0.0193	0.0379	−0.0016	0.0308	0.0010	0.9956	0.9976	1.0037	1.0082	1.0454	1.1159
Mean	0.0060	0.0199	0.0094	−0.0099	0.0039	0.0001	0.9997	0.9956	1.0481	0.9744	0.9981	0.9981
Median	0.0054	0.0193	0.0094	−0.0027	−0.0033	0.0032	0.9956	0.9987	1.0298	0.9981	0.9974	1.0011
SD	0.0083	0.0150	0.0259	0.0192	0.0225	0.0136	0.0269	0.0389	0.0743	0.0485	0.0598	0.0634

Abbreviations: Natl, national-scale kriging; MN, kriging in middle north region; NE, kriging in northeast region; NW, kriging in northwest region; SE, kriging in southeast region; SW, kriging in southwest region.

## Appendix 1 WHI Institutions and Investigators

**Table:**

WHI Program Office, National Heart, Lung, and Blood Institute, Bethesda, MD	Barbara Alving, Jacques Rossouw, Shari Ludlam, Linda Pottern, Joan McGowan, Leslie Ford, Nancy Geller
Clinical Coordinating Centers	
Fred Hutchinson Cancer Research Center, Seattle, WA	Ross Prentice, Garnet Anderson, Andrea LaCroix, Charles L. Kooperberg, Ruth E. Patterson, Anne McTiernan, Shirley Beresford
Wake Forest University School of Medicine, Winston-Salem, NC	Sally Shumaker
Medical Research Labs, Highland Heights, KY	Evan Stein
University of California–San Francisco, San Francisco, CA	Steven Cummings
Clinical Centers	
Albert Einstein College of Medicine, Bronx, NY	Sylvia Wassertheil-Smoller
Baylor College of Medicine, Houston, TX	Jennifer Hays
Brigham and Women’s Hospital, Harvard Medical School, Boston, MA	JoAnn Manson
Brown University, Providence, RI	Annlouise R. Assaf
Emory University, Atlanta, GA	Lawrence Phillips
George Washington University Medical Center, Washington, DC	Judith Hsia
Harbor-UCLA Research and Education Institute, Torrance, CA	Rowan Chlebowski
Kaiser Permanente Center for Health Research, Portland, OR	Evelyn Whitlock
Kaiser Permanente Division of Research, Oakland, CA	Bette Caan
Medical College of Wisconsin, Milwaukee, WI	Jane Morley Kotchen
MedStar Research Institute/Howard University, Washington, DC	Barbara V. Howard
Northwestern University, Chicago/Evanston, IL	Linda Van Horn
Ohio State University, Columbus, OH	Rebecca Jackson
Rush Medical Center, Chicago, IL	Henry Black
Stanford Prevention Research Center, Stanford, CA	Marcia L. Stefanick
State University of New York–Stony Brook, Stony Brook, NY	Dorothy Lane
University of Alabama at Birmingham, Birmingham, AL	Cora E. Lewis
University of Arizona, Tucson/Phoenix, AZ	Tamsen Bassford
University at Buffalo, Buffalo, NY	Jean Wactawski-Wende
University of California–Davis, Sacramento, CA	John Robbins
University of California–Irvine, Irvine, CA	F. Allan Hubbell
University of California–Los Angeles, Los Angeles, CA	Howard Judd
University of California–San Diego, La Jolla/Chula Vista, CA	Robert D. Langer
University of Cincinnati, Cincinnati, OH	Margery Gass
University of Florida, Gainesville/Jacksonville, FL	Marian Limacher
University of Hawaii, Honolulu, HI	David Curb
University of Iowa, Iowa City/Davenport, IA	Robert Wallace
University of Massachusetts/Fallon Clinic, Worcester, MA	Judith Ockene
University of Medicine and Dentistry of New Jersey, Newark, NJ	Norman Lasser
University of Miami, Miami, FL	Mary Jo O’Sullivan
University of Minnesota, Minneapolis, MN	Karen Margolis
University of Nevada, Reno, NV	Robert Brunner
University of North Carolina–Chapel Hill, Chapel Hill, NC	Gerardo Heiss
University of Pittsburgh, Pittsburgh, PA	Lewis Kuller
University of Tennessee, Memphis, TN	Karen C. Johnson
University of Texas Health Science Center, San Antonio, TX	Robert Brzyski
University of Wisconsin, Madison, WI	Gloria E. Sarto
Wake Forest University School of Medicine, Winston-Salem, NC	Denise Bonds
Wayne State University School of Medicine/Hutzel Hospital, Detroit, MI	Susan Hendrix
